# The Effect of Handwashing with Water or Soap on Bacterial Contamination of Hands

**DOI:** 10.3390/ijerph8010097

**Published:** 2011-01-06

**Authors:** Maxine Burton, Emma Cobb, Peter Donachie, Gaby Judah, Val Curtis, Wolf-Peter Schmidt

**Affiliations:** Department of Infectious and Tropical Diseases, London School of Hygiene and Tropical Medicine, Keppel Street, London, WC1E 7HT, UK; E-Mails: m_burton5@hotmail.com (M.B.); Emma.Cobb@lshtm.ac.uk (E.C.); Peter.Donachie@lshtm.ac.uk (P.D.); Gaby.Judah@lshtm.ac.uk (G.J.); Val.Curtis@lshtm.ac.uk (V.C.)

**Keywords:** hygiene, trial, infection

## Abstract

Handwashing is thought to be effective for the prevention of transmission of diarrhoea pathogens. However it is not conclusive that handwashing with soap is more effective at reducing contamination with bacteria associated with diarrhoea than using water only. In this study 20 volunteers contaminated their hands deliberately by touching door handles and railings in public spaces. They were then allocated at random to (1) handwashing with water, (2) handwashing with non-antibacterial soap and (3) no handwashing. Each volunteer underwent this procedure 24 times, yielding 480 samples overall. Bacteria of potential faecal origin (mostly *Enterococcus* and *Enterobacter* spp.) were found after no handwashing in 44% of samples. Handwashing with water alone reduced the presence of bacteria to 23% (p < 0.001). Handwashing with plain soap and water reduced the presence of bacteria to 8% (comparison of both handwashing arms: p < 0.001). The effect did not appear to depend on the bacteria species. Handwashing with non-antibacterial soap and water is more effective for the removal of bacteria of potential faecal origin from hands than handwashing with water alone and should therefore be more useful for the prevention of transmission of diarrhoeal diseases.

## 1. Introduction

Diarrhoeal diseases are one of the leading causes of child death around the world [1]. The World Health Organisation (WHO) recognises the spread of diarrhoeal diseases as a serious global problem [2] and estimates that each year, there are more than 2.2 million lives lost due to these infections, more than from malaria, HIV/AIDS and measles combined [1]. The majority of these deaths are in children under 5 years of age [3]. It has been suggested that handwashing may substantially reduce the risk of diarrhoeal diseases [4].

Promotion of improved hand hygiene has been recognised as an important public health measure but it is unclear how much hand hygiene is required to interrupt transmission of diarrhoea pathogens. In particular it has not been conclusively shown whether use of soap is essential to remove pathogens from hands. Recent hygiene promotion campaigns especially in low income settings have not been unanimous in recommending soap use [4].

A number of studies have compared different hand hygiene methods in hospital settings [5]. In contrast, few studies have been published on the effect of hand hygiene on bacterial contamination of hands in the community. Hoque and colleagues found that a wide variety of hand cleansing means in poor settings (soap, ash, mud) are effective in reducing the contamination with coliform bacteria on hands [6,7]. In a small randomised trial the same author reported that soap may be more effective than water in reducing the presence of coliform bacteria on hands [6].

Luby and colleagues found that a simple microbiological method with three fingers directly imprinting a MacConkey agar for thermotolerant coliforms was unable to distinguish between households who were given soap during a large randomized handwashing trial and control households [8]. They concluded that the method was unsuitable for the evaluation of handwashing practices. However, the lack of difference in bacterial contamination may have been due to lack of compliance with the intervention. We thought that a proof-of-principle trial was needed where participants would be given specific tasks to contaminate their hands in a naturalistic setting and where handwashing was done under supervision.

We conducted a randomised controlled trial to determine whether non-antibacterial soap is better at reducing bacteria of potential faecal origin than water only. A further goal was to clarify whether a simple microbiological test that can be applied to large groups in a relatively short time [9,10] would be able to distinguish people who practice handwashing from those who don’t.

## 2. Experimental Section

This study was carried out between July and August 2009. Overall, 20 volunteers were taken to a large, frequently visited British museum, or asked to travel on a bus or the underground. They were asked to deliberately wipe their hands over hand contact surfaces such as handrails, door handles and seats with the aim of contaminating their hands with whatever bacteria were present. Using a pre-determined random sequence, not known to the participants during self-contamination, participants were then asked to wash their hands with soap, to use water only or not to wash at all. Each volunteer underwent this sequence 24 times, 8 times for each of the three hand hygiene approaches (soap, water, no handwash). Participants assigned to handwashing were asked to wash their hands as they would normally do, without instructions on length of time or thoroughness. The volunteers allocated to handwashing were then provided with a paper towel to dry their hands. A wet NaCl-soaked charcoal swab was then wiped across the fingers of the dominant hand of the participant. The participants were finally given an alcohol gel to clean their hands (78% total alcohol content, Ethanol 71% / Propanol 29%, Softalind Viscorub, Braun-Melsungen). The swabs were returned to the laboratory within 5 hours of being taken. In total, 480 samples were collected; 160 after handwashing with plain soap, 160 after handwashing with water alone and 160 with no handwashing. During the experimental phase we measured the amount of time taken to conduct handwashing with and without soap, once for each volunteer.

Upon arrival at the laboratory the swabs were immediately cut into a universal tube containing 10 mL of Purple MacConkey broth using aseptic techniques. The swabs were incubated at 35 °C for 48 hours. All samples were then streaked onto the MacConkey agar No.3 and Bile Aesculin agar. MacConkey agar No. 3 is a selective media which can differentiate between coliforms and non-lactose fermenters, whilst inhibiting gram-positive cocci. These plates were incubated for 18–24 hours at 35 °C. For all other colonies produced on MacConkey agar No. 3 and those which were spot indole negative, a gram stain, catalase and oxidase test was carried out followed by an API 20E biochemical test to determine the identity of the bacteria. Bile Aesculin agar is a differential medium for the isolation of *Enterococcus* spp. and group D *Streptococcus* and inhibition of other gram positive bacteria. These plates were incubated at 37 °C for 18–24 hours. *Enterococcus* and Group D *Streptococcus* spp. are able to hydrolyse the aesculin to form aesculetin, producing a brown/black complex. Any white colonies on Bile Aesculin agar were presumed to be *Staphylococcus* spp. and any black colonies were tested with Lancefield group D antisera. Agglutination indicated a positive result for *Enterococcus* spp.

The prevalence of bacterial contamination in the three study arms (soap, water, no handwash) was compared using logistic regression. Since the same volunteers repeatedly underwent testing, within-subject correlation was accounted for by the use of generalised estimating equations (GEE) with robust standard errors. If the cell numbers were too low for conducting regression analysis, Fishers exact test was used instead, ignoring clustering (the design effect was found to be low, see results).

## 3. Results and Discussion

**Figure 1:**
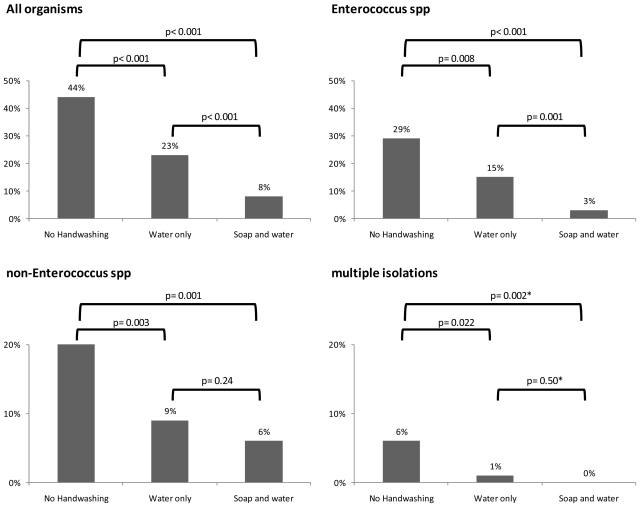
Effect of handwashing with water alone or soap and water compared to no handwashing. P-values derived from logistic regression adjusted for within-person correlation, except * where p-value was derived from Fishers exact test ignoring within-person correlation. The design effect due to within-person clustering was low (around 1.2–1.3). Note different y-axis scales in top *vs.* bottom panels.

Table 1 shows the different organisms isolated in the three study arms. *Enterococcus* spp. were the most common bacteria found, followed by *Enterobacter* spp. Figure 1 shows the effect of handwashing with soap or water only on contamination, compared to no handwashing. Overall, handwashing with water alone reduced the prevalence of bacteria substantially. Handwashing with soap was more effective in reducing the prevalence of contamination and specifically of *Enterococcus* spp. There was a trend that handwashing with soap was also more effective in reducing the prevalence of other species and of multiple isolates, but the statistical support was low (Figure 1).

The effect of repeated measurements in the same individual was low: the design effect (the factor by which a sample size needs to be increased to achieve the same statistical power as an unclustered study) ranged from 1.2 to 1.3 (depending on the comparison group).

Participants were asked to wash their hands as long and as thorough as they would normally do. The length of time required to carry out handwashing was measured once for each method in all volunteers. Participants took on average 12 seconds (standard deviation 2.8) to wash their hands with water alone, and 14 seconds (standard deviation 2.3) to wash their hands with water and soap (p = 0.02).

Thus, handwashing with soap took them only slightly longer than handwashing with water alone. It seems unlikely that this small difference can explain the large difference in the removal of bacteria. Soap on its own appears to have an effect on the removal of bacteria of potential faecal origin, independent of the possibility that soap use may cause people to wash their hands longer.

Unlike the study by Hoque and colleagues our trial was conducted in an experimental (albeit naturalistic) setting, where volunteers deliberately contaminated their hands. Additional testing showed that this approach increased the prevalence of contamination from around 10% to over 40% of individuals. It also improved control over the conduct of the experiment, but may affect generalisability, as the study primarily aimed at providing a proof of principle. However, we believe that the superior effect of soap on the removal of bacteria compared to water alone as the principal finding of our study is unlikely to depend on the setting.

Not all of the bacteria isolated in our study are known to cause disease in humans. Surprisingly, we found few E. coli on hands which may be due to their short survival time in the environment. Overall, the effect of soap appeared to be independent of the type of bacteria (Figure 1), a view which is supported by the study by Hoque and colleagues who found a similar effect of hand hygiene on unspecified faecal coliform bacteria [6]. However, the power of our study to detect differences between species was low.

We used plain non-antibacterial soap for the experiment. Future studies could address whether antibacterial soap is more effective in removing pathogens from hands. However, Luby and colleagues conducted a large double-blind randomised trial in Pakistan and found antibacterial soap no more effective in reducing diarrhoea than normal soap [11]. It is still not clear whether or in what circumstances anti-bacterial soaps offer a health advantage [12].

The bacteriological methods used in this study provide no quantification of bacterial load, unlike a study by Hoque and colleagues [7]. Quantifying the effect of different hand washing procedures on bacterial load may be particularly helpful for studies in poor settings with poor sanitation facilities, where the environmental contamination with faecal organisms is much higher [13–15]. We also tested a semi-quantitative finger-print method used previously in Thailand [15] not unsimilar to the method used by Luby and colleagues [8] but found that contamination levels were too low to provide consistent results. Therefore we decided to use a qualitative method.

It seems reasonable to assume that handwashing with soap is also more effective in reducing bacterial load compared to water alone. Future studies could address the effect of different hand hygiene procedures on removing gastro-intestinal or respiratory viruses such as influenza A. Hands have been implicated especially in the spread of Norovirus [16]. Viral studies are more difficult to conduct as viruses may not be as present in the environment as often as are bacteria of faecal origin, but they may be possible for example if patients with laboratory confirmed infection are recruited as volunteers. Alternatively, healthy volunteers may experimentally contaminate their hands with cultured viruses before undergoing different hand hygiene regimes, as was done in a recent study on influenza A H1N1 [17]. This study found that handwashing with soap was better at removing influenza A H1N1 than several hand sanitizers. Handwashing with water alone was not tested.

## 4. Conclusions

The results demonstrate that handwashing with non-antibacterial soap is much more effective in removing bacteria from hands than handwashing with water only. Although handwashing with water alone reduced the presence of bacteria on hands substantially, the study supports the policy of many current hand hygiene campaigns promoting the use of soap [18,19]. The strong association between hand hygiene method and bacterial contamination of hands found in our study suggests that the prevalence of faecal indicator bacteria may also be used to monitor changes in hygiene behaviour in the general population, for example following hygiene promotion campaigns.

Hygiene behaviour is difficult to measure because people tend to change their behaviour under observation or over-report desired practices [15,20]. We have previously shown that our test kit can be used to study associations between hygiene relevant behaviours and hand contamination [9]. We found that test results positive for bacteria of potential faecal origin were more common in people frequently shaking hands, reporting soil contact or those scoring low on a hygiene score based on self-report [9]. The microbiological method used in this and our earlier studies [9,10] is relatively simple and of low cost (around $3.80). Its suitability for large scale use in the evaluation of handwashing campaigns in low income settings where handwashing should be most beneficial remains to be investigated. A sophisticated laboratory infrastructure may not be required to conduct testing. However, modifying the method to allow semi-quantitative or quantitative analysis may be necessary if contamination rates are high [15].

## Figures and Tables

**Table 1:** Organisms found after self-contamination of hands, and handwashing with either soap, water only, or no handwashing.

Faecal Bacteria	No Handwashing	Water only	Soap and water
*Enterococcus* spp.	46 (29%)	24 (15%)	4 (3%)
*Enterobacter amnigenus*	14 (9%)	4 (3%)	4 (3%)
*Enterobacter cloacae*	13 (8%)	5 (3%)	2 (1%)
*Shigella* spp.	2 (1%)	1 (1%)	0 (0%)
*Klebsiella* spp.	5 (3%)	2 (1%)	1 (1%)
*E. coli* spp.	0 (0%)	0 (0%)	1 (1%)
*Pantoea* spp.	0 (0%)	2 (1%)	1 (1%)

Multiple isolations	10 (6%)	2 (1%)	0 (0%)
Any bacteria	70 (44%)	36 (23%)	13 (8%)

Total	160 (100%)	160 (100%)	160 (100%)

## References

[1] Boschi-Pinto C Velebit L Shibuya K Estimating child mortality due to diarrhoea in developing countries Bull. WHO 2008 86 710 707 18797647 10.2471/BLT.07.050054 PMC2649491

[2] WHO Health Statistics 2008, Mortality and Burden of Disease WHO Geneva, Switzerland 2008 Available online: http://www.who.int/whosis/whostat/EN_WHS08_Table1_Mort.pdf (accessed on 16 November 2010).

[3] Black RE Morris SS Bryce J Where and why are 10 million children dying every year? Lancet 2003 361 2226 2234 12842379 10.1016/S0140-6736(03)13779-8

[4] Ejemot RI Ehiri JE Meremikwu MM Critchley JA Hand washing for preventing diarrhoea Cochrane Database Syst. Rev 2008 1 Art. No. CD004265 10.1002/14651858.CD004265.pub2 18254044

[5] WHO Guidelines on Hand Hygiene in Health Care WHO Geneva, Switzerland 2009 Available online: http://whqlibdoc.who.int/publications/2009/9789241597906_eng.pdf (accessed on 16 November 2010).

[6] Hoque BA Briend A A comparison of local handwashing agents in Bangladesh J. Trop. Med. Hyg 1991 94 61 64 1995938

[7] Hoque BA Mahalanabis D Alam MJ Islam MS Post-defecation handwashing in Bangladesh: practice and efficiency perspectives Public Health 1995 109 15 24 7871142 10.1016/s0033-3506(95)80071-9

[8] Luby SP Agboatwalla M Billhimer W Hoekstra RM Field trial of a low cost method to evaluate hand cleanliness Trop. Med. Int. Health 2007 12 765 771 17550474 10.1111/j.1365-3156.2007.01847.x

[9] Dodrill L Schmidt WP Cobb E Donachie P Curtis V de Barra M Male commuters in North and South England: Risk factors for the presence of faecal bacteria on hands BMC Public Health 2010 (in press) 10.1186/1471-2458-11-31 PMC3031219 21226924

[10] Judah G Donachie P Cobb E Schmidt W Holland M Curtis V Dirty hands: bacteria of faecal origin on commuters’ hands Epidemiol. Infect 2010 138 409 414 19723362 10.1017/S0950268809990641

[11] Luby SP Agboatwalla M Feikin DR Painter J Billhimer W Altaf A Hoekstra RM Effect of handwashing on child health: A randomised controlled trial Lancet 2005 366 225 233 16023513 10.1016/S0140-6736(05)66912-7

[12] Aiello AE Larson EL Levy SB Consumer antibacterial soaps: Effective or just risky? Clin. Infect. Dis 2007 45 Suppl 2 S137 S147 17683018 10.1086/519255

[13] Hoque BA Mahalanabis D Pelto B Alam MJ Research methodology for developing efficient handwashing options: An example from Bangladesh J. Trop. Med. Hyg 1995 98 469 475 8544234

[14] Kaltenthaler EC Drasar BS Potter CW The use of microbiology in the study of hygiene behaviour Microbios 1996 88 35 43 9121378

[15] Pinfold JV Horan NJ Measuring the effect of a hygiene behaviour intervention by indicators of behaviour and diarrhoeal disease Trans. Roy. Soc. Trop. Med. Hyg 1996 90 366 371 8882177 10.1016/s0035-9203(96)90507-6

[16] Barker J Vipond IB Bloomfield SF Effects of cleaning and disinfection in reducing the spread of Norovirus contamination via environmental surfaces J. Hosp. Infect 2004 58 42 49 15350713 10.1016/j.jhin.2004.04.021

[17] Grayson ML Melvani S Druce J Barr IG Ballard SA Johnson PD Mastorakos T Birch C Efficacy of soap and water and alcohol-based hand-rub preparations against live H1N1 influenza virus on the hands of human volunteers Clin. Infect. Dis 2009 48 285 291 19115974 10.1086/595845

[18] Curtis V Sidibe M Scott BE lyer P Sara J The Handwash Handbook: A Guide for Developing a Hygiene Promotion Program to Increase Handwashing with Soap The World Bank Group Washington, DC, USA 2005 67 68

[19] Scott BE Schmidt WP Aunger R Garbrah-Aidoo N Animashaun R Marketing hygiene behaviours: The impact of different communication channels on reported handwashing behaviour of women in Ghana Health Educ. Res 2008 23 392 401 18000025 10.1093/her/cym056

[20] Biran A Rabie T Schmidt W Juvekar S Hirve S Curtis V Comparing the performance of indicators of hand-washing practices in rural Indian households Trop. Med. Int. Health 2008 13 278 285 18304276 10.1111/j.1365-3156.2007.02001.x

